# Multi-System Langerhans Cell Histiocytosis as a Mimic of IgG4-Related Disease: A Case Report and Literature Review

**DOI:** 10.3389/fendo.2022.896227

**Published:** 2022-07-22

**Authors:** Xiaohui Feng, Lu Zhang, Fuqiong Chen, Gang Yuan

**Affiliations:** Department of Endocrinology, Tongji Hospital, Huazhong University of Science and Technology, Wuhan, China

**Keywords:** Langerhans cell histiocytosis, central diabetes insipidus, diagnosis, IgG4-related disease, case report

## Abstract

Langerhans cell histiocytosis (LCH) is a rare disease characterized by the clonal accumulation and/or proliferation of specific dendritic cells resembling normal epidermal Langerhans cells (LCs). Clinical manifestations are variable, depending on the affected tissues or organs, however, LCH with elevated serum IgG4 has not been reported. Herein, we reported a 26-year-old Chinese female multi-system LCH (MS-LCH) who first presented with central diabetes insipidus (CDI), accompanied by panhypopituitarism and hepatic dysfunction. Diagnostic investigations were strongly suspicious of IgG4-RD because of elevated serum IgG4 levels during the process. Furtherly, thyroid and lymph node involvement and biopsy led to the diagnosis of MS-LCH; the strongly positive staining of CD1a, S100, CD207 (langerin), and Ki67 was found. Moreover, after systemic treatment with five cycles of chemotherapy, many lesions were greatly improved. Since both LCH and IgG4-RD are orphan diseases that can affect any organ, the differential diagnosis is challenging, especially when LCH is associated with unexplained serum IgG4 elevation. In this article, the case of a young woman suffering from MS-LCH that affected organs including the pituitary, thyroid, lymph node, and liver was summarized, and relevant literature was reviewed to better equip the diagnosis and treatment in its early stages.

## Introduction

The etiology of LCH is unknown and may be a clonal proliferation of LCH cells similar to LCs with local or extensive infiltration in any organ (1, 2). The disease is heterogeneous, varying from single bone lesions to a multi-system disease with organ failure and substantial mortality. Thus, the classification of LCH includes single system LCH (SS-LCH) and MS-LCH (3, 4). LCH can affect any organ of the body, but those more frequently affected are the bones (70%-80% of cases), skin (30%-50%), liver, spleen, hematopoietic system, or lungs (30% each) and the endocrine system involvement including pituitary and thyroid is quite rare (5–8). If the patient has hypothalamic-pituitary involvement, diabetes insipidus (DI) is the typical manifestation (9). In patients with central diabetes insipidus and pituitary stalk thickening on imaging studies, LCH should be considered in the differential diagnosis. We herein report an unusual case of elevated IgG4 multisystem adult LCH, with involvement of the pituitary gland, the thyroid gland, lymph node, and liver to add further information to the diagnosis and treatment of this challenging disease.

## Case Presentation

A 26-year-old Chinese female first presented with polyuria and polydipsia in November 2017. At that time, her daily drinking volume reached almost 5L, which was equivalent to the urine volume, accompanied by dry skin, fatigue, lethargy, decreased vision, and about 2kg weight loss. The patient’s impaired liver function was reflected by elevated alanine aminotransferase (ALT) (121U/L, normal range 0-40U/L) and aspartate aminotransferase (AST) levels (74U/L, normal range 0-40U/L). All hormone levels were within normal ranges, except for elevated prolactin (PRL) (41.15ng/ml, normal range 3.34-26.72ng/mL). Pituitary Magnetic Resonance Imaging (MRI) scan exhibited that the posterior pituitary hyperintensity displayed local enhancement that was lower than the anterior lobe and the pituitary stalk was thickened (Figure not shown). There was no family history of central diabetes insipidus or panhypopituitarism. After diagnosis of DI, the patient was administered desmopressin (0.2mg/d), and the symptoms went into remission. The timeline is shown to better follow the case from symptom onset to final treatment (**Supplementary Figure S1**).

Then, half a year later, the patient manifested with menstrual disorders, menstrual volume gradually decreased, accompanied with severe mood disorders, memory loss, anorexia, fatigue, difficulty falling asleep, and weight loss (a drop of nearly 8kg). The urine routine was normal at this time. Subsequently, hormonal tests revealed that the levels of follicle stimulating hormone (FSH), luteinizing hormone (LH), estrogen (E2), progesterone (P), and testosterone (T) were below what is normal for adult women (**Table 1**). In addition, the level of PRL increased to 142.3ng/ml. After reexamination of the pituitary MRI scan, the results showed pituitary stalk-hypothalamus nodular thickening, larger than the front, which lead to the consideration of possible tumorous lesions or lymphocytic hypophysitis; the pituitary itself became thinner than the front, and the posterior lobe was not clearly displayed (Figure not shown). However, it is important to note that the serum immunoglobulin G4 (IgG4) level was highly elevated, up to 1.7g/L; the cutoff value is >1.35g/L. Meanwhile, the ratio of IgG4 to IgG increased to 15.60%. The diagnosis led to lymphocytic hypophysitis. Without biopsy, the patient only received hormone replacement therapy including glucocorticoid, proglucone, estradiol, progesterone, thyroxine, and desmopressin. The patient’s symptoms were relieved.

**Table 1:** Hormone profiles of the patient.

Parameter	June 2018	Nov. 2019	Reference range
FSH	1.14	1.37	F:3.5-12.5; O:4.7-21.5;
			L:1.7-7.7; M:25.8-134.8IU/L
LH	<0.20	<0.20	F:2.4-12.6; O:14.0-95.6;
			L:1.0-11.4; M:7.7-58.5IU/L
Estradiol	<5	29	F:12.5-166; O:85.8-498;
			L:43.8-211; M: <5.0-54.7pg/mL
Progesterone	<0.05	0.82	F:0.2-1.5; O:0.8-3.8;
			L:1.7-27; M:0.1-0.8ng/mL
Testosterone	<0.025	0.24	0.1-0.75ng/mL
Prolactin	142.3	52.07	4.79-28.3ng/mL
SHBG	103.2	34.5	26.1-100nmol/L
ACTH(8AM)	1.74	0.53	1.1-11.0pmol/L
Cortisol(8AM)	33.80	34.53	42-248ug/L
Cortisol(4PM)	33.12	33.07	29-173ug/L
Cortisol(12MN)	16.40	32.47	0-67ug/L
TSH	0.349	0.604	0.27–4.2uIU/mL
fT3	3.02	2.66	2.0–4.4pg/mL
fT4	10.98	13.28	9.32–17.09pg/mL

FSH, Follicle-Stimulating Hormone; LH, Luteinizing Hormone; SHBG, sex hormone-binding globulin; F, follicular phase; O, ovulatory phase; L, luteal phase; M, menopause; ACTH, adrenocorticotropin; TSH, thyroid stimulating hormone; fT3, free triiodothyronine; fT4, free thyroxine.

Because the patient stopped glucocorticoid and thyroxine for more than half a year, and estradiol was also stopped 1 month prior to her admission to the hospital the patient readmitted to hospital in June 2019. Two months before admission, the patient experienced fatigue, poor appetite, lethargy, accompanied by polyuria and polydipsia. Physical examination revealed that several enlarged lymph nodes were palpable in the neck and the thyroid gland was II° enlarged. Blood routine showed increased neutrophils (6.95×10^9/L, normal range 1.80×10^9-6.30×10^9/L) and platelets (412.0×10^9/L, normal range 100×10^9-300×10^9/L). Erythrocyte Sedimentation Rate (ESR) raised up to 86mm/H (normal range 0-20mm/H) and hypersensitive C-reactive protein (hs-CRP) also increased to 69.9mg/L (normal range 0-8mg/L). Liver function abnormality was shown by increased AST levels 69U/L, γ-glutamyl transpeptidase 168U/L (normal range, 6-42U/L), alkaline phosphatase (ALP) 286U/L (normal range, 35-105U/L), and direct bilirubin (DBIL) 13.2umol/L (normal range 0-6.8umol/L). Meanwhile, APTT, PT, FIB, and TT were all elevated, which reflected a clotting disorder. The level of IgG4 still increased to 4.280g/L. However, hormone levels were back to normal.

**Figure 1:**
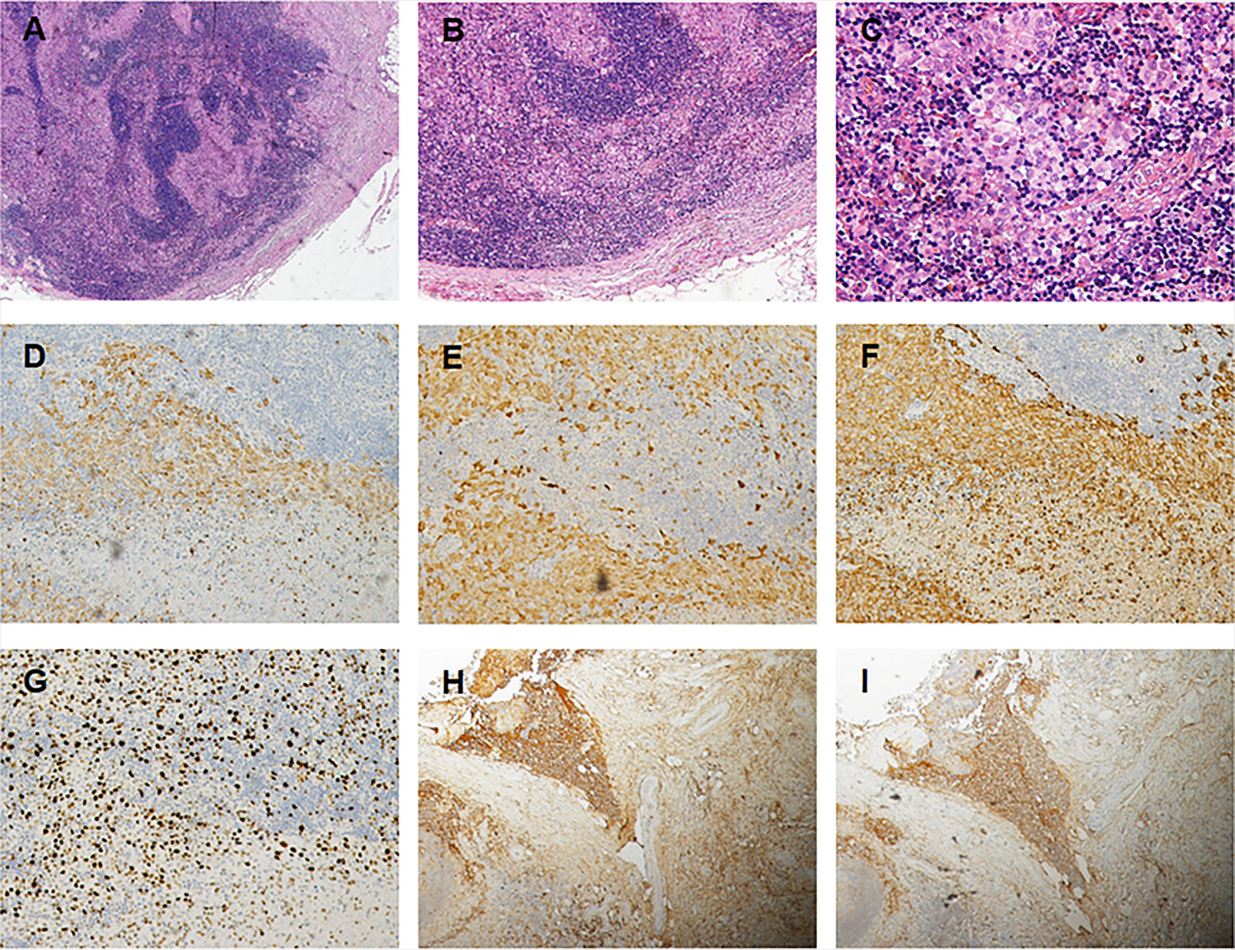
**(A)** Photomicrograps of a low power image showing an inflammatory in filtrate composed of Langerhans cells lymphocytes (Hex 4). **(B)** The clusters of Langerhans cells are accompanied by lymphocytes and an occasional eosinophil (HE x10). **(C)** The nuclear morphology exhibited by the Langerhan cells, which have relatively uniform was positive to Langerin **(D)**, S-100 protein **(E)** and to CD1a **(F)**, and Ki–67 **(G)** antigen markers (x 20). Immunostains for IgH **(H)** and IgG4 **(I)** show an IgG4/IgG ratio <40 %, some immune cells were positive and stained, but no positive cells were found in the Langerhans (x 10).

**Figure 2:**
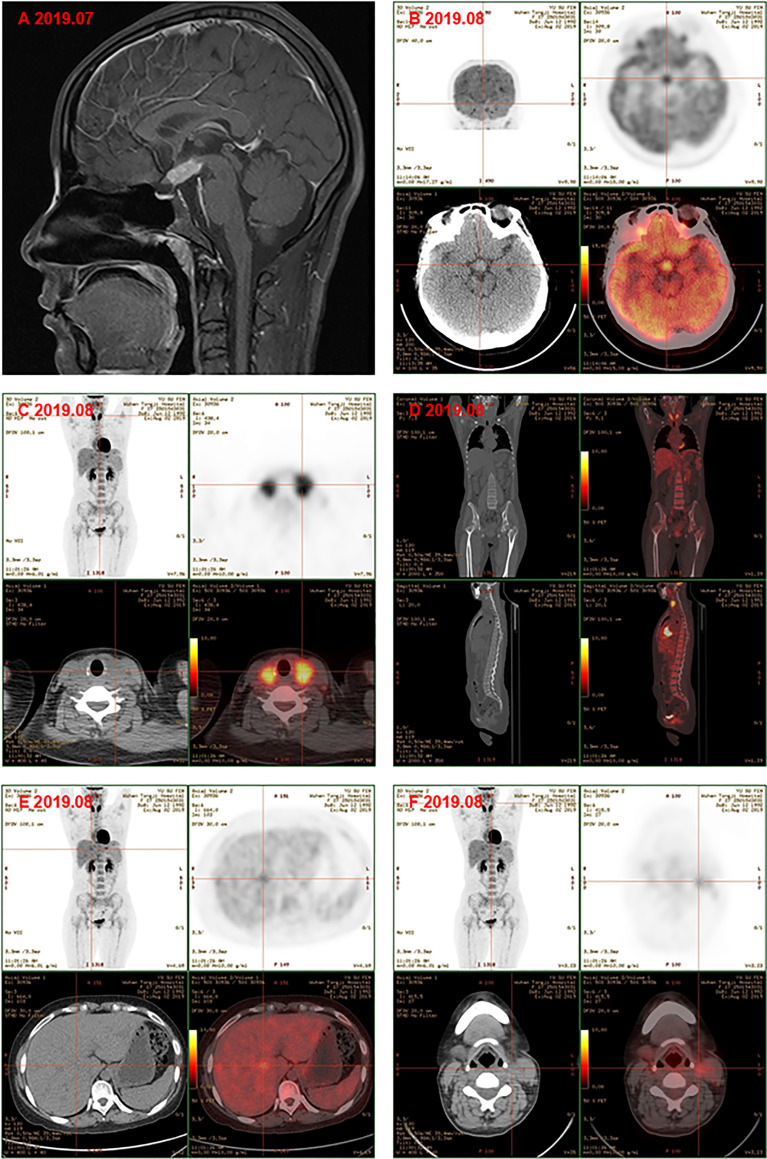
**(A)** Magnetic resonance image of pituitary gland showd the nodular thickening and enhancement of the hyphothalamus, and the pituitary itself became thinner. PET-CT showed the metabolism of hypothalamus was higher **(B)**; the volume of bilobe thyroid and metabolism increased **(C)**; multiple lymph nodes icreased and metabolism increased, bone marrow metabolism slightly increased **(D)**; multiple slightly low density shadows in the liver **(E)**; bilateral tonsil metabolism increased **(F)** before chemotherapy.

Subsequently, color doppler ultrasound of the thyroid and lymph nodes revealed a diffuse heterogeneous thyroid enlargement with slightly rich blood flow and multiple enlarged lymph nodes on both sides of the neck. To further clarify the goiter, the patient underwent a thyroid and lymph node biopsy. The results suggested an epithelial neoplasm, with heavy eosinophilic infiltration (**Figures 1A–C)**, for which immunohistochemistry showed that the Langerhans cells expressed CD207 (langerin) (**Figure 1D**), S-100 (**Figure 1E**), CD1a (**Figure 1F**), and Ki67 (**Figure 1G**), but were negative for IgG4 (**Figure 1H**) and IgG (**Figure 1I**). MRI of the pituitary suggested nodular thickening and enhancement of the hypothalamus (**Figure 2A**). Under the supervision of a specialist in hematology, tumor metabolic imaging (PET/CT) was performed, and the results were as follows: 1. Nodular thickening of hypothalamus and increased metabolism (**Figure 2B**); 2. The volume of bilobed thyroid increased and metabolism increased (**Figure 2C**); 3. Multiple lymph nodes increased (bilateral neck, mediastinum paratracheal, hilar region, portal space, retroperitoneum, bilateral iliac vascular), increased metabolism (**Figure 2D**); 4. Multiple slightly low density shadows in the liver, increased uneven metabolism (**Figure 2E**); 5. Bilateral tonsil metabolism increased, and bone marrow metabolism uneven slightly increased (**Figure 2F**).

**Figure 3:**
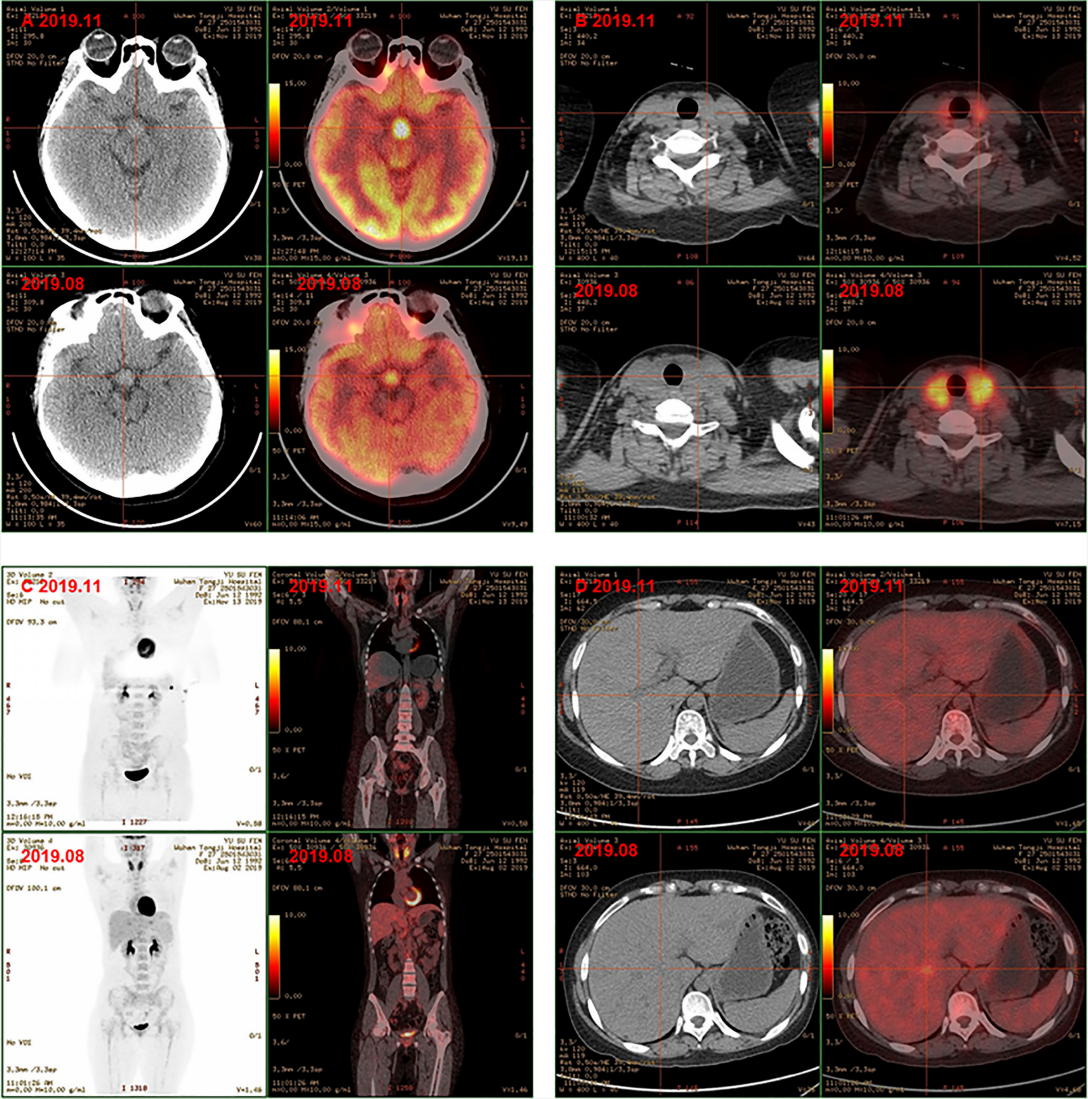
PET-CT showed hyphothalamic nodule was thickened and the metabolism of hypothalamus was higher **(A)**; the volume and metabolism of thyroid gland decreased **(B)**; the range of primary lesions and the metabolism was significantly reduced, the metabolism of bilateral tonsils and bone marrow was similar to the previoud **(C)**; metabolism of the multiple foci in the liver subsided **(D)** after chemotherapy.

Finally, the clinical picture and the biopsy findings were consistent with a diagnosis of MS-LCH with pituitary, thyroid, lymph node, and liver involvement. The patient was then given DVP chemotherapy (vinblastine 4mg day1, cyclophosphamide 600mg day1, prednisone 10mg days1-5). In addition, hormone replacement therapy and antidepressant therapy were continued. After three cycles, favorable evolution was noted, with a good tolerance of treatment and a clear relief of results of PET/CT examination. Comparison with previous PET/CT (**Figures 2B–F)**: 1. Hypothalamic nodule was thickened, and slightly larger than before; the metabolism of hypothalamus was higher (**Figure 3A**); 2. The volume and metabolism of thyroid gland decreased (**Figure 3B**); 3. The range of primary lesions (neck, mediastinum, and abdominal lymph nodes) was significantly reduced, and the metabolism was significantly reduced/subsided (**Figure 3C**); 4. Metabolism of the multiple foci in the liver subsided (**Figure 3D**); 5. The metabolism of bilateral tonsils and bone marrow was increased, which was similar to the previous (**Figure 3C**). Given this, we upped the dose of DVP (vinblastine 4mg day1, cyclophosphamide 1000mg day1, prednisone 10mg days (1-5) in the fourth chemotherapy cycle.

After the fourth chemotherapy cycle, the symptoms of polyuria, polydipsia, and menstrual disorders were improved. The patient had a positive attitude to the treatment she received, but became anxious for the long diagnosis and treatment process at times.

## Discussion

LCH is caused by the clonal proliferation of myeloid precursor cells, which differentiate into CD1a^+^/CD207^+^ cells in the lesions, resulting in a series of organ involvement and dysfunction (10). Its overall incidence is reported to be one to two in a million, and it is rarer in adults (9). However, various degrees of systemic organ involvement can occur in all ages. The disease was first described by Paul Langerhans in 1868 (11). It was initially thought that the pathogenesis of this disease was the reactive clonal proliferation of Langerhans cells (LCS), but now more and more scholars believe that it is an inflammatory myeloid tumor (10, 12, 13). Adult LCH is also considered likely to represent the manifestation of an aberrant immune response to an unspecified antigenic stimulus rather than a manifestation of tumor proliferation. The growing understanding of the pathogenesis of the disease has opened a new chapter for targeted therapies.

The clinical manifestations of LCH are diverse; almost all tissues may be involved with or without associated dysfunction (14–16). Therefore, a full systematic evaluation with PET-CT is usually required when this diagnosis is suspected. As reported in this case, diabetes insipidus is usually one of the earliest manifestations of the disease, indicating a predisposition to hypothalamic/pituitary axis involvement (9, 17, 18). A multicenter study showed that diabetes insipidus occurred in 24% of patients and was the most common permanent consequence of LCH (19). Further PET examination revealed that in addition to pituitary involvement, there were many other organs affected, including the thyroid, multiple lymph nodes, lungs, liver, and bone marrow throughout the body, which indicated multiple endocrine organs were involved in this systemic disease. Up to 40% of patients with LCH experience pulmonary manifestations with nodular or reticular pattern observed on chest radiograph as typical lesions. Thus, chest CT can be pivotal for the diagnosis. The formation of early-stage granulomas in the lung is easy to observe. However, nodule formation in other organs, especially solid organs, can be difficult to detect on imaging, despite the similar nature of the lesions (20). Owing to the rarity of LCH in adults, coupled with its nonspecific and diverse clinical presentation, it often leads to missed and delayed diagnosis (3, 21). Since pituitary imaging examination suggested nodular thickening of pituitary stalk to hypothalamus, this patient was initially misdiagnosed as lymphocytic hypophysitis and central diabetes insipidus.

Interestingly, we found an unexplained increase in serum IgG4 levels. Therefore, when the patient was readmitted, we considered the diagnosis as IgG4-related disease (IgG4-RD), which is also a multisystem disease. At this point, the differential diagnosis of the disease is particularly important. Both LCH and IgG4-RD can be characterized by multisystemic involvement and an extremely variable clinical course, which usually progresses over months to years, with lesions often appearing metachronously. IgG4-RD is an immune-mediated fibro-inflammatory disease, which can also affect multiple organs, and patients often present with subacute mass formation in the affected organs (e.g., orbital pseudo tumor, renal mass similar to renal cell carcinoma, nodular lesion of lung) or diffuse enlargement of the organs (e.g., pancreas) (22). In 60%-90% of patients with IgG4-RD, multiple organs are involved, and different diseases are named according to different organs involved, such as type 1 (IgG4-related) autoimmune pancreatitis (AIP), Riedel thyroiditis (IgG4-related thyroid disease), IgG4-related pituitaritis, etc. (23–26). The hallmark histologic characteristics of IgG4-RD is dense lymphoplasmacytic infiltration dominated by IgG4+ plasma cells in the affected tissue, often accompanied by a certain degree of fibrosis, phlebitis obliterans, and eosinophilic granulocytosis (27). Diagnosis of IgG4-RD depends on histopathological biopsy (28). In this case, many manifestations can be misled to a diagnosis of IgG4-related pituitaritis and thyroiditis, like higher serum IgG4 with thyroid and lymph node enlargement, combined with MRI showing enlargement of the pituitary gland and the pituitary stalk. Only after the thyroid and cervical lymph node biopsy and the pathological results showing Langerhans cells, local cell hyperplasia, and immunohistochemical display CD1a, CD207, according to positive, no IgG4+ plasma cells of dense lymphatic plasma cell infiltration, can the diagnosis of LCH be proven. To further complicate the issue, it should be emphasized that high levels of IgG4 in the serum could not be sufficient for diagnosing an IgG4-RD since an elevation of IgG4 may occur in patients harboring pathologic conditions other than IgG4-RD (29). Since no similar cases have been reported before, the reason why serum IgG4 concentrations were elevated in this case remains unknown. Maybe this case is a manifestation of an aberrant immune response to an unspecified antigenic stimulus, which participated in the formation of IgG4.

Currently, treatment for LCH usually includes immunosuppressive regimen, chemotherapy, or radiotherapy. However, treatment for adult patients has not been standardized (3). Chemotherapies based on vinblastine and steroids are often the first line of choice. Compared with the toxic effects of traditional chemotherapy drugs, indomethacin with lower side effects is gradually becoming a new first-line treatment drug (30–32).

Our study has some limitations. Firstly, we did not detect the BRAF V600E mutation in this patient. Studies have reported that a mutation in BRAF V600E was found in 38%-64% of LCH cases, and it has been proven that this mutation is associated with adverse pathological conditions and high risks (33–35). Secondly, as of the writing of the article, after chemotherapy, we found that the patient’s hypothalamus still had high metabolism, and the final clinical outcome of the patient has not been observed.

Given the knowledge acquired from this case report, though the morbidity of MS-LCH is very low, we should keep it in mind constantly in clinical practice, especially in cases with diabetes insipidus as one of the earliest manifestations. In face of a similar patient in the future, MS-LCH should be considered as a differential diagnosis. Diagnostic imaging (routine radiography, CT, and MRI) plays important roles in the diagnosis, monitoring, and evaluation of treatment outcomes for patients with this disease. Biopsy of an involved organ without delay is needed to make an earlier definite diagnosis and to use treatment to prevent MS-LCH in time.

In conclusion, adult LCH is a relatively rare disease, its pathogenesis is not yet clear, clinical manifestations are diverse, and missed diagnosis and misdiagnosis are common. This is the first report of an adult female patient with central diabetes insipidus as the initial symptom, accompanied by elevated serum IgG4, who was eventually diagnosed with LCH by thyroid and lymph node biopsy. To point out clinical significance, reliance upon serum IgG4 concentrations to diagnose IgG4-RD is less rigorous. Multiple non-IgG4-RD conditions can result in elevated serum IgG4 concentrations. Careful clinical examination and basic laboratory investigations may provide a comparatively accurate cause. However, the gold standard for diagnosis in most cases remains biopsy of an involved organ and close clinicopathologic correlation of the findings. This case report highlights the clinical, laboratory, and imaging signs in multi-system LCH that should be highlighted to help doctors recognize, diagnose, and treat similar cases more quickly.

## Data Availability Statement

The original contributions presented in the study are included in the article/**Supplementary Material**. Further inquiries can be directed to the corresponding author.

## Ethics Statement

The studies involving human participants were reviewed and approved by Ethics Committee, Tongji Hospital of Tongji Medical College, Huazhong University of Science and Technology. The patients/participants provided their written informed consent to participate in this study. Written informed consent was obtained from the individual(s) for the publication of any potentially identifiable images or data included in this article.

## Author Contributions

GY conceived and designed the study. XF and LZ performed the experiments and wrote the paper. FC and GY reviewed and edited the manuscript. All authors read and approved the manuscript.

## Funding

This work was supported by the National Natural Science Foundation of China (81974121).

## Conflict of Interest

The authors declare that the research was conducted in the absence of any commercial or financial relationships that could be construed as a potential conflict of interest.

## Publisher’s Note

All claims expressed in this article are solely those of the authors and do not necessarily represent those of their affiliated organizations, or those of the publisher, the editors and the reviewers. Any product that may be evaluated in this article, or claim that may be made by its manufacturer, is not guaranteed or endorsed by the publisher.

## References

[1] Vassallo R Ryu JH Colby TV Hartman T Limper AH. Pulmonary Langerhans'-Cell Histiocytosis. N Engl J Med (2000) 342(26):1969–78. doi: 10.1056/NEJM200006293422607 10877650

[2] Goyal G Young JR Koster MJ Tobin WO Vassallo R Ryu JH. The Mayo Clinic Histiocytosis Working Group Consensus Statement for the Diagnosis and Evaluation of Adult Patients With Histiocytic Neoplasms: Erdheim-Chester Disease, Langerhans Cell Histiocytosis, and Rosai-Dorfman Disease. Mayo Clin Proc (2019) 94(10):2054–71. doi: 10.1016/j.mayocp.2019.02.023 31472931

[3] Girschikofsky M Arico M Castillo D Chu A Doberauer C Fichter J. Management of Adult Patients With Langerhans Cell Histiocytosis: Recommendations From an Expert Panel on Behalf of Euro-Histio-Net. Orphanet J Rare Dis (2013) 8:72. doi: 10.1186/1750-1172-8-72 23672541 PMC3667012

[4] Emile JF Abla O Fraitag S Horne A Haroche J Donadieu J. Revised Classification of Histiocytoses and Neoplasms of the Macrophage-Dendritic Cell Lineages. Blood (2016) 127(22):2672–81. doi: 10.1182/blood-2016-01-690636 PMC5161007 26966089

[5] Arico M. Langerhans Cell Histiocytosis in Adults: More Questions Than Answers? Eur J Cancer (2004) 40(10):1467–73. doi: 10.1016/j.ejca.2004.01.025 15196529

[6] Makras P Stathi D Yavropoulou M Tsoli M Kaltsas G. The Annual Incidence of Langerhans Cell Histiocytosis Among Adults Living in Greece. Pediatr Blood Cancer (2020) 67(9):e28422. doi: 10.1002/pbc.28422 32618036

[7] Radzikowska E. Pulmonary Langerhans' Cell Histiocytosis in Adults. Adv Respir Med (2017) 85(5):277–89. doi: 10.5603/ARM.a2017.0046 29083024

[8] Kobayashi M Tojo A. Langerhans Cell Histiocytosis in Adults: Advances in Pathophysiology and Treatment. Cancer Sci (2018) 109(12):3707–13. doi: 10.1111/cas.13817 PMC6272080 30281871

[9] Makras P Alexandraki KI Chrousos GP Grossman AB Kaltsas GA. Endocrine Manifestations in Langerhans Cell Histiocytosis. Trends Endocrinol Metab (2007) 18(6):252–7. doi: 10.1016/j.tem.2007.06.003 17600725

[10] Rodriguez-Galindo C Allen CE. Langerhans Cell Histiocytosis. Blood (2020) 135(16):1319–31. doi: 10.1182/blood.2019000934 32106306

[11] Langerhans P. Ueber Die Nerven Der Menschlichen Haut. Haut Archiv f Pathol Anat 44(1868):325–37. doi: 10.1007/BF01959006

[12] Arico M Egeler RM. Clinical Aspects of Langerhans Cell Histiocytosis. Hematol Oncol Clin North Am (1998) 12(2):247–58. doi: 10.1016/S0889-8588(05)70508-6 9561898

[13] Willman CL Busque L Griffith BB Favara BE McClain KL Duncan MH. Langerhans'-Cell Histiocytosis (Histiocytosis X)–A Clonal Proliferative Disease. N Engl J Med (1994) 331(3):154–60. doi: 10.1056/NEJM199407213310303 8008029

[14] Haupt R Minkov M Astigarraga I Schafer E Nanduri V Jubran R. Langerhans Cell Histiocytosis (LCH): Guidelines for Diagnosis, Clinical Work-Up, and Treatment for Patients Till the Age of 18 Years. Pediatr Blood Cancer. (2013) 60(2):175–84. doi: 10.1002/pbc.24367 PMC4557042 23109216

[15] Ni M Yang X. Langerhans' Cell Histiocytosis of the Temporal Bone: A Case Report. Exp Ther Med (2017) 13(3):1051–3. doi: 10.3892/etm.2017.4072 PMC5403409 28450940

[16] Li X Deng QI Li YM. A Case of Langerhans' Cell Histiocytosis Following Hodgkin's Disease. Mol Clin Oncol (2016) 5(1):27–30. doi: 10.3892/mco.2016.860 27330759 PMC4906978

[17] Makras P Samara C Antoniou M Zetos A Papadogias D Nikolakopoulou Z. Evolving Radiological Features of Hypothalamo-Pituitary Lesions in Adult Patients With Langerhans Cell Histiocytosis (LCH). Neuroradiology (2006) 48(1):37–44. doi: 10.1007/s00234-005-0011-x 16292545

[18] Kaltsas GA Powles TB Evanson J Plowman PN Drinkwater JE Jenkins PJ. Hypothalamo-Pituitary Abnormalities in Adult Patients With Langerhans Cell Histiocytosis: Clinical, Endocrinological, and Radiological Features and Response to Treatment. J Clin Endocrinol Metab (2000) 85(4):1370–6. doi: 10.1210/jcem.85.4.6501 10770168

[19] Haupt R Nanduri V Calevo MG Bernstrand C Braier JL Broadbent V. Permanent Consequences in Langerhans Cell Histiocytosis Patients: A Pilot Study From the Histiocyte Society-Late Effects Study Group. Pediatr Blood Cancer. (2004) 42(5):438–44. doi: 10.1002/pbc.20021 15049016

[20] Cong CV Ly TT Duc NM. Multisystem Langerhans Cell Histiocytosis: Literature Review and Case Report. Radiol Case Rep (2022) 17(5):1407–12. doi: 10.1016/j.radcr.2022.02.024 PMC8891995 35251425

[21] Pierro J Vaiselbuh SR. Adult Langerhans Cell Histiocytosis As a Diagnostic Pitfall. J Clin Oncol (2016) 34(6):e41–5. doi: 10.1200/JCO.2013.50.3045 24958830

[22] Khosroshahi A Stone JH. A Clinical Overview of IgG4-Related Systemic Disease. Curr Opin Rheumatol (2011) 23(1):57–66. doi: 10.1097/BOR.0b013e3283418057 21124086

[23] Haraguchi A Era A Yasui J Ando T Ueki I Horie I. Putative IgG4-Related Pituitary Disease With Hypopituitarism and/or Diabetes Insipidus Accompanied With Elevated Serum Levels of Igg4. Endocr J (2010) 57(8):719–25. doi: 10.1507/endocrj.K10E-030 20467161

[24] Shimatsu A Oki Y Fujisawa I Sano T. Pituitary and Stalk Lesions (Infundibulo-Hypophysitis) Associated With Immunoglobulin G4-Related Systemic Disease: An Emerging Clinical Entity. Endocr J (2009) 56(9):1033–41. doi: 10.1507/endocrj.K09E-277 19926920

[25] Sah RP Chari ST Pannala R Sugumar A Clain JE Levy MJ. Differences in Clinical Profile and Relapse Rate of Type 1 Versus Type 2 Autoimmune Pancreatitis. Gastroenterology (2010) 139(1):140–8. doi: 10.1053/j.gastro.2010.03.054 20353791

[26] Falhammar H Juhlin CC Barner C Catrina SB Karefylakis C Calissendorff J. Riedel's Thyroiditis: Clinical Presentation, Treatment and Outcomes. Endocrine (2018) 60(1):185–92. doi: 10.1007/s12020-018-1526-3 PMC5845586 29380231

[27] Perugino CA Stone JH. IgG4-Related Disease: An Update on Pathophysiology and Implications for Clinical Care. Nat Rev Rheumatol (2020) 16(12):702–14. doi: 10.1038/s41584-020-0500-7 32939060

[28] Deshpande V Zen Y Chan JK Yi EE Sato Y Yoshino T. Consensus Statement on the Pathology of IgG4-Related Disease. Mod Pathol (2012) 25(9):1181–92. doi: 10.1038/modpathol.2012.72 22596100

[29] Carruthers MN Khosroshahi A Augustin T Deshpande V Stone JH. The Diagnostic Utility of Serum IgG4 Concentrations in IgG4-Related Disease. Ann Rheum Dis (2015) 74(1):14–8. doi: 10.1136/annrheumdis-2013-204907 24651618

[30] De Benedittis D Mohamed S Rizzo L Santopietro M Palumbo G Cardarelli L. Indomethacin is an Effective Treatment in Adults and Children With Bone Langerhans Cell Histiocytosis (LCH). Br J Haematol (2020) 191(5):e109–e13. doi: 10.22541/au.159377589.99736276 32862434

[31] Munn SE Olliver L Broadbent V Pritchard J. Use of Indomethacin in Langerhans Cell Histiocytosis. Med Pediatr Oncol (1999) 32(4):247–9. doi: 10.1002/(SICI)1096-911X(199904)32:4<247::AID-MPO1>3.0.CO;2-J 10102016

[32] Han I Suh ES Lee SH Cho HS Oh JH Kim HS. Management of Eosinophilic Granuloma Occurring in the Appendicular Skeleton in Children. Clin Orthop Surg (2009) 1(2):63–7. doi: 10.4055/cios.2009.1.2.63 PMC2766756 19885056

[33] Badalian-Very G Vergilio JA Degar BA MacConaill LE Brandner B Calicchio ML. Recurrent BRAF Mutations in Langerhans Cell Histiocytosis. Blood (2010) 116(11):1919–23. doi: 10.1182/blood-2010-04-279083 PMC3173987 20519626

[34] Haroche J Cohen-Aubart F Emile JF Arnaud L Maksud P Charlotte F. Dramatic Efficacy of Vemurafenib in Both Multisystemic and Refractory Erdheim-Chester Disease and Langerhans Cell Histiocytosis Harboring the BRAF V600E Mutation. Blood (2013) 121(9):1495–500. doi: 10.1182/blood-2012-07-446286 23258922

[35] Huang H Lu T Sun Y Li S Li J Xu K. Association Between Clinicopathologic Characteristics and BRAF(V600E) Expression in Chinese Patients With Langerhans Cell Histiocytosis. Thorac Cancer (2019) 10(10):1984–92. doi: 10.1111/1759-7714.13179 PMC6775012 31441596

