# Supplementary material for: Multi-System Langerhans Cell Histiocytosis as a Mimic of IgG4-Related Disease: A Case Report and Literature Review
Source: Front Endocrinol (Lausanne). 2022 Jul 22;13:896227. doi: 10.3389/fendo.2022.896227 (PMC9353717; doi:10.3389/fendo.2022.896227)
Supplement: Supplementary file 1 [file DataSheet_1.pdf]

CARE
case report guidelines

CARE Checklist of information to include when writing a case report

(cc) BY-NC-ND

Topic	Item	Checklist item description	Reported on Line
Title	1	The diagnosis or intervention of primary focus followed by the words "case report"	page1, line1
Key Words	2	2 to 5 key words that identify diagnoses or interventions in this case report, including "case report"	page1, line25-26
Abstract (no references)	3a	Introduction:What is unique about this case and what does it add to the scientific literature?	page1, line10-11
	3b	Main symptoms and/or important clinical findings	page1, line12-14
	3c	The main diagnoses,therapeutic interventions,and outcomes	page1, line14-19
	3d	Conclusion—What is the main "take-away" lesson(s)from this case?	page1, line21-24
Introduction	4	One or two paragraphs summarizing why this case is unique (**may include references**)	page1, line28-41
Patient Information	5a	De-identified patient specific information.	page2, line2
	5b	Primary concerns and symptoms of the patient.	page2, line3-5
	5c	Medical, family, and psycho-social history including relevant genetic information	page2, line12
	5d	Relevant past interventions with outcomes	page2, line12-14
Clinical Findings	6	Describe significant physical examination (PE) and important clinical findings.	page2, line19-29
Timeline	7	Historical and current information from this episode of care organized as a timeline	page9, table1
Diagnostic Assessment	8a	Diagnostic testing (such as PE, laboratory testing, imaging, surveys).	page3, line4-20
	8b	Diagnostic challenges (such as access to testing, financial, or cultural)	no applicable
	8c	Diagnosis (including other diagnoses considered).	page3, line21-22
	8d	Prognosis (such as staging in oncology)where applicable	no applicable
Therapeutic Intervention	9a	Types of therapeutic intervention (such as pharmacologic,surgical,preventive,self-care).	page3, line23
	9b	Administration of therapeutic intervention (such as dosage,strength,duration).	page3, line22-25
	9c	Changes in therapeutic intervention (with rationale).	page3, line34-35
Follow-up and Outcomes	10a	Clinician and patient-assessed outcomes (if available).	page3, line25-27
	10b	Important follow-up diagnostic and other test results	page3, line27-33
	10c	Intervention adherence and tolerability (How was this assessed?).	page3, line37-38
	10d	Adverse and unanticipated events	no applicable
Discussion	11a	A scientific discussion of the strengths AND limitations associated with this case report	page5, line21-26
	11b	Discussion of the relevant medical literature **with references**.	page3, line40-page4, line27
	11c	The scientific rationale for any conclusions (including assessment of possible causes).	page4, line28-page5, line13
	11d	The primary "take-away" lessons of this case report (without references)in a one paragraph conclusion	page5, line27-34
Patient Perspective	12	The patient should share their perspective in one to two paragraphs on the treatment(s)they received	page3, line37-38
Informed Consent	13	Did the patient give informed consent? Please provide if requested	Yes ☑ No ☐
