# Supplementary figures and images for: Multi-System Langerhans Cell Histiocytosis as a Mimic of IgG4-Related Disease: A Case Report and Literature Review
Source: Front Endocrinol (Lausanne). 2022 Jul 22;13:896227. doi: 10.3389/fendo.2022.896227 (PMC9353717; doi:10.3389/fendo.2022.896227)

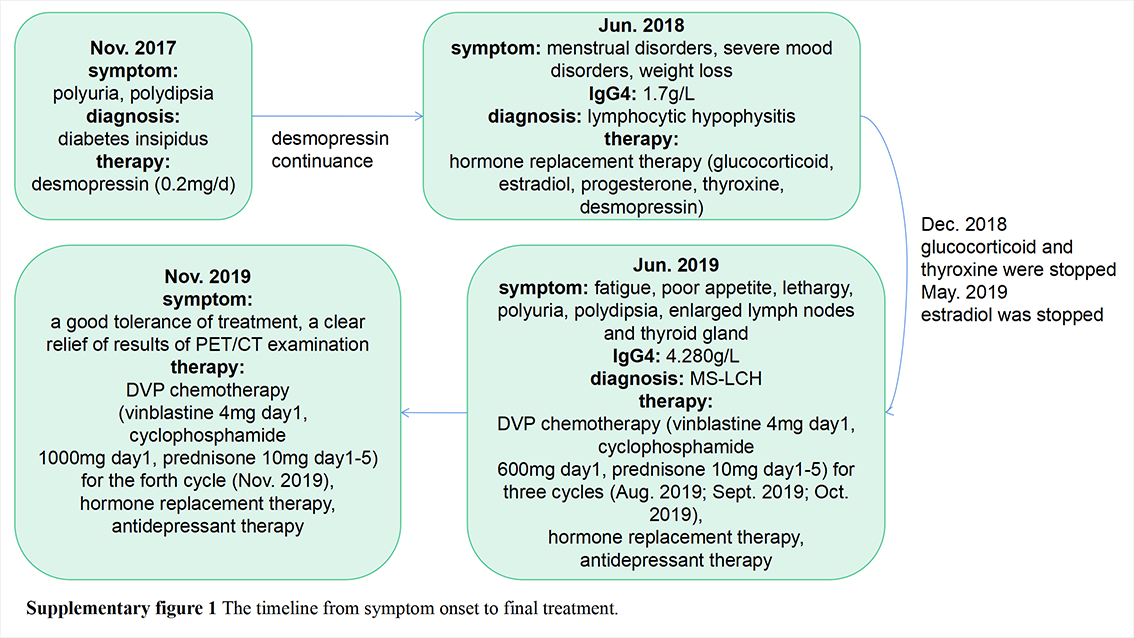

Supplement: Supplementary file 2 [file Image_1.tif]
